# Vaccine-Preventable Infectious Disease Mortality Among Children in the Post-vaccine Era: Persistent Burden and Emerging Temporal Trends

**DOI:** 10.7759/cureus.114702

**Published:** 2026-08-17

**Authors:** Sabina Nisar, Syamily Parambath, Banita Negi, Mohtashim Jameel, Harmandeep Kaur, Harshpreet Singh, Rithik Dharan

**Affiliations:** 1 Pediatric Surgery, Sher-i-Kashmir Institute of Medical Sciences, Srinagar, IND; 2 Pediatrics, Srinivasan Medical College and Hospital, Tiruchirappalli, IND; 3 Pediatrics, Government Doon Medical College and Hospital, Dehradun, IND; 4 Pediatrics, Fatima Hospital, Hyderabad, IND; 5 Internal Medicine, Star Hospital, Jalandhar, IND; 6 Pharmacology, Tamil Nadu Dr. M.G.R. Medical University, Chennai, IND

**Keywords:** cdc wonder, childhood mortality, influenza, joinpoint regression, vaccine-preventable infectious diseases

## Abstract

Background: Despite remarkable reductions in vaccine-preventable infectious diseases following the introduction of routine childhood immunization, preventable childhood deaths continue to occur. This study evaluated temporal trends, disease-specific mortality patterns, and demographic disparities in childhood mortality due to vaccine-preventable infectious diseases over 25 years.

Methods: A nationwide retrospective population-based study was conducted using mortality data from the Centers for Disease Control and Prevention Wide-ranging Online Data for Epidemiologic Research (CDC WONDER) Multiple Cause of Death databases. Deaths among children younger than 18 years attributed to vaccine-preventable infectious diseases between 1999 and 2024 were identified using the International Classification of Diseases, 10th Revision codes. Temporal trends were assessed using Joinpoint regression, while negative binomial regression was used to estimate adjusted mortality rate ratios for demographic and geographic factors.

Results: A total of 5,520 deaths due to vaccine-preventable infectious diseases were identified, corresponding to an overall crude mortality rate of 0.29 per 100,000 children. Mortality declined initially but subsequently fluctuated without a sustained downward trend. Influenza accounted for 60.7% of all deaths and was the only disease demonstrating a significant increase in mortality over time. Significant declines were observed in pneumococcal disease mortality, meningococcal disease mortality, and infant mortality, whereas mortality among children aged five to nine years increased significantly. Negative binomial regression demonstrated significantly higher adjusted mortality rate ratios among American Indian or Alaska Native and Black or African American children compared with White children.

Conclusion: Childhood mortality due to vaccine-preventable infectious diseases remains an important public health concern despite substantial advances in immunization. Influenza emerged as the predominant and only disease with an increasing mortality trend, while persistent racial and geographic disparities highlight continuing inequities in disease burden. Strengthening routine and catch-up immunization programs, improving equitable access to vaccination, and maintaining robust surveillance are essential to further reduce preventable childhood deaths.

## Introduction

Vaccination is considered one of the most successful public health programs ever. According to the World Health Organization (WHO), vaccination saves 3.5-5 million lives each year from diseases like diphtheria, tetanus, whooping cough, flu, and measles [1,2]. Childhood immunization has led to a drastic reduction in cases, morbidity, and mortality associated with vaccine-preventable infectious diseases (VPIDs) in children [3,4]. The introduction of the Vaccines for Children (VFC) initiative has increased access to childhood vaccines and saved around 508 million disease cases, 32 million hospitalizations, and 1.129 million deaths in children born from 1994 to 2023 [5].

Even though vaccinations have significantly reduced deaths from infections that can be prevented by vaccines, vaccine-preventable deaths continue to occur. This may be caused by lack of adequate vaccination coverage, delays in vaccination, waning immunity against certain pathogens, differences in healthcare quality, and epidemiology of infections [6-8]. Babies are especially prone to infection-related deaths since they cannot receive the full dose of vaccines due to their age and therefore depend mostly on passive immunity provided by maternal antibodies in the early stages of life [9]. Collectively, these factors demonstrate that the availability of effective vaccines alone is insufficient to eliminate vaccine-preventable mortality and underscore the importance of continued mortality surveillance to identify populations that remain at greatest risk. Although disease incidence and vaccination coverage are routinely monitored, mortality surveillance is useful in determining the long-term efficacy of vaccination programs by identifying populations that are still experiencing mortality and evaluating changes in disease epidemiology [10].

Despite great progress in surveillance of vaccine-preventable diseases, there is a need for continuous surveillance of long-term mortality in order to assess the lasting effects of immunization programs, detect changing patterns in disease epidemiology, detect disadvantaged populations, and make recommendations for evidence-based vaccination policy [10]. However, systematic evaluation of long-term mortality trends associated with multiple childhood vaccine-preventable infectious diseases, with consideration of demographic and geographic differences in these patterns, is rare.

Therefore, the primary objective of this nationwide retrospective population-based study was to evaluate temporal trends in mortality due to vaccine-preventable infectious diseases among children aged <18 years in the United States from 1999 to 2024 using the Centers for Disease Control and Prevention Wide-ranging Online Data for Epidemiologic Research (CDC WONDER) Multiple Cause of Death database. Secondary objectives were to describe disease-specific mortality patterns, age-specific mortality trends, and demographic and geographic disparities to identify populations experiencing a persistent burden of vaccine-preventable infectious disease mortality and to inform future public health and pediatric immunization strategies.

## Materials and methods

Study design

A nationwide retrospective population-based epidemiological study was conducted to evaluate mortality from vaccine-preventable infectious diseases (VPIDs) among children <18 years of age during the period from January 1999 to December 2024. The study evaluated long-term temporal patterns in VPID mortality across the United States using routinely collected mortality data, assessed disease-specific mortality trends to identify changes in the burden of individual vaccine-preventable infectious diseases over the study period, and examined differences in mortality across demographic and geographic subgroups to identify populations experiencing a persistent burden of VPID mortality.

Data source

Mortality data for the United States were obtained from the Centers for Disease Control and Prevention Wide-ranging Online Data for Epidemiologic Research (CDC WONDER) Multiple Cause of Death databases, which are based on death certificate information compiled by the National Vital Statistics System (NVSS). To cover the complete study period (1999-2024), data from the Multiple Cause of Death, 1999-2020 database (bridged-race categories) were combined with data from the Multiple Cause of Death by Single Race, 2018-2024 database. Because the two CDC WONDER databases overlap during 2018-2020, deaths from 1999 to 2017 were extracted from the bridged-race database, whereas deaths from 2018 to 2024 were obtained exclusively from the Multiple Cause of Death by Single Race database. This approach avoided duplicate inclusion of records from the overlapping years. Vaccine-preventable infectious disease deaths corresponding to diseases included in the routine childhood immunization schedule in the United States were identified using the underlying cause of death coded according to the International Classification of Diseases, 10th Revision (ICD-10). Annual population estimates available through CDC WONDER were used as the denominator to calculate crude mortality rates. The study period began in 1999, corresponding to the nationwide implementation of ICD-10 mortality coding in the United States [11,12]. The full list of ICD-10 codes is presented in Table 1.

**Table 1:** List of ICD-10 codes used. ICD-10: International Classification of Diseases, 10th Revision

Disease	ICD-10 code(s)
Influenza	J09-J11
Meningococcal disease	A39
Pertussis (whooping cough)	A37
Hepatitis A	B15
Varicella	B01
Measles	B05
Mumps	B26
Rubella	B06
Diphtheria	A36
Tetanus	A33-A35
Pneumococcal disease	J13 (pneumococcal pneumonia), G00.1 (pneumococcal meningitis), A40.3 (sepsis due to *Streptococcus pneumoniae*)
*Haemophilus influenzae* type b disease (Hib)	G00.0 (Haemophilus meningitis), J14 (pneumonia due to *Haemophilus influenzae*), A41.3 (sepsis due to *Haemophilus influenzae*)

Study population and case definition

All deaths among individuals younger than 18 years who died of a VPID as the underlying cause of death between 1999 and 2024 were included in the study. Individuals aged ≥18 years and records of unspecified age were excluded from the analyses. Diseases with suppressed number of deaths (<10 deaths) were included in the analysis as a whole but could not be separately analyzed due to restrictions of CDC WONDER.

Cases were selected through ICD-10 codes for vaccine-preventable infections, which are part of the routine vaccination schedule in childhood, such as influenza, meningococcal infection, pneumococcal infection, pertussis, hepatitis A, *Haemophilus influenzae* infection, varicella, measles, mumps, rubella, diphtheria, and tetanus. Certain vaccine-preventable diseases, such as hepatitis B, poliomyelitis, and rotavirus infection, were not included because they are infrequent causes of childhood mortality and are not consistently identifiable as underlying causes of death for population-based mortality trend analysis using the selected ICD-10 case definition.

Study variables

The main outcome measure was mortality from VPIDs, described as the annual number of deaths and crude mortality rate per 100,000 children. The independent variables included age groups (below one year, one to four years, five to nine years, 10-14 years, and 15-17 years), gender, race (White, Black or African American, Asian or Pacific Islander, and the American Indian {AI} or the Alaska Native {AN}), the United States Census region (Northeast, Midwest, South, and West), and urban-rural status by the National Center for Health Statistics 2013 Urban-Rural Classification Scheme [13]. Calendar years were classified into five periods (1999-2003, 2004-2008, 2009-2013, 2014-2018, and 2019-2024).

Statistical analysis

Categorical variables were described by frequency and percentage, while mortality was reported as annual death counts and crude mortality rate per 100,000 children. Age-adjusted mortality rate was not computed since our analyses were limited only to the pediatric population, and age-related trends were analyzed independently.

Joinpoint Regression Program version 6.0.1 (Bethesda, MD: National Cancer Institute) was configured to allow a maximum of three joinpoints. The optimal model was selected using the Monte Carlo permutation test with a significance level of 0.05 according to the default model selection criteria of the National Cancer Institute Joinpoint Regression Program. Annual percent changes (APCs) and their 95% confidence intervals (CIs) were obtained by applying log-linear regression models. Statistically significant changes in trends were detected via the Monte Carlo permutation method.

The relationship between study period and mortality from specific causes was examined using Pearson's chi-square test. Negative binomial regression was used to identify which demographic and geographic factors were associated with mortality, since mortality counts demonstrated overdispersion. Mortality rate ratios (MRRs) and 95% CIs were calculated using population size as the offset variable. Missing data were not imputed. All statistics other than Joinpoint analysis were done using IBM SPSS Statistics version 27.0 for Windows (Armonk, NY: IBM Corp.). All tests were two-tailed, and p<0.05 was used as the cutoff level for statistical significance.

Ethical considerations

The current study was based on the use of publicly available de-identified data from the CDC WONDER database. Ethics committee approval or patient consent was not required because the study did not involve any identifiable patient data or participation. The study complied with the Declaration of Helsinki.

## Results

**Figure 1:**
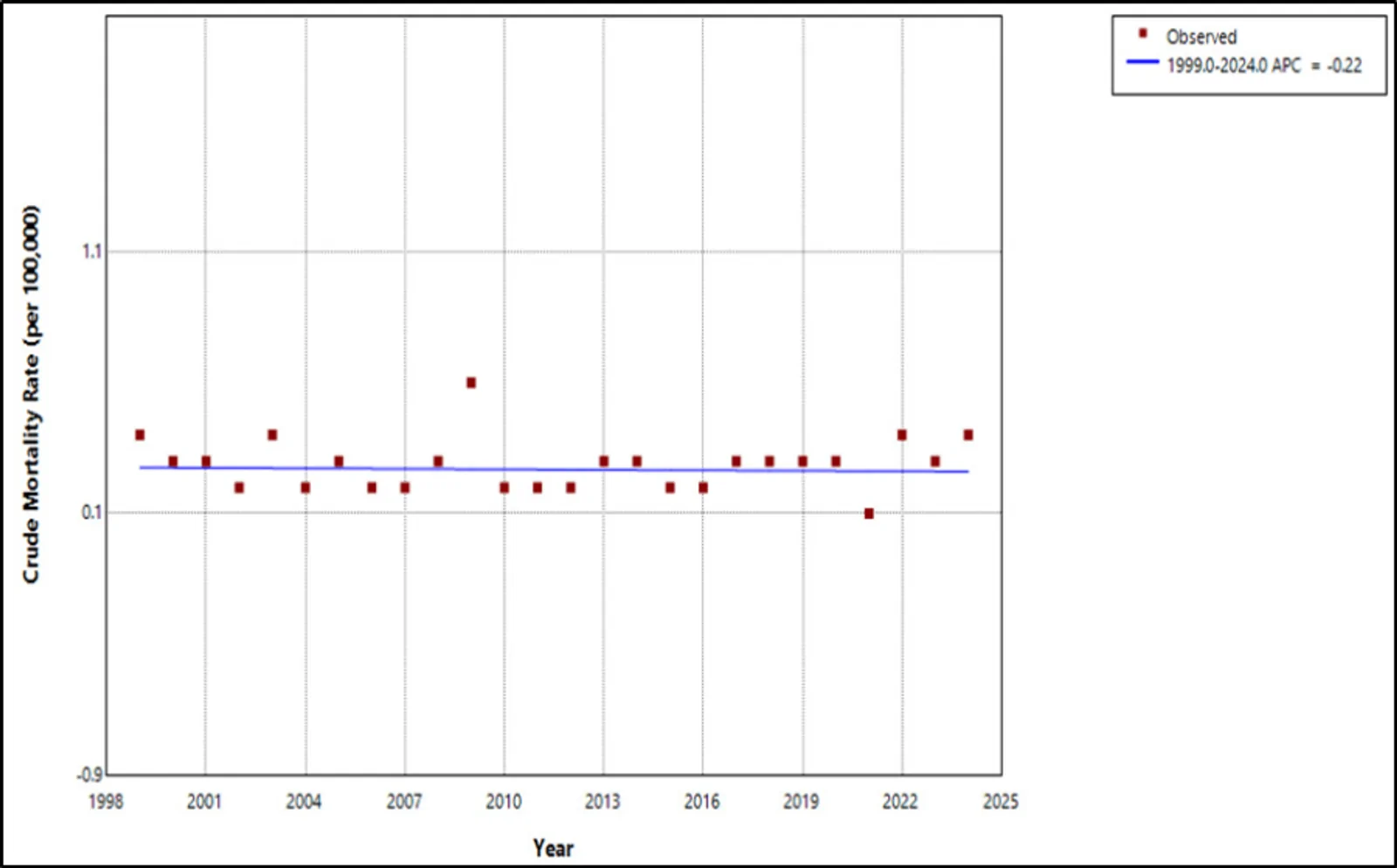
Joinpoint regression analysis of overall vaccine-preventable disease mortality among children. Observed annual crude mortality rates (red squares) and the fitted Joinpoint regression trend (blue line) for vaccine-preventable infectious disease mortality among children in the United States from 1999 to 2024. No Joinpoint was identified during the study period, indicating a single overall trend with an APC of -0.22. Mortality rates are expressed per 100,000 population. APC: annual percent change

The overall mortality rates due to vaccine-preventable infectious diseases in children over the study period are shown in Table 2 and Figure 1. Over the period from 1999 to 2024, there were a total of 5,520 deaths due to vaccine-preventable infectious diseases in children younger than 18 years, with an overall crude mortality rate of 0.29 per 100,000 population. The crude mortality rate was highest during the period from 1999 to 2003 (0.33 per 100,000), after which the crude mortality rate declined to 0.25 per 100,000 during the period from 2004 to 2008. Subsequently, the crude mortality rates ranged from 0.26 to 0.31 per 100,000 over the other study periods. The highest number of deaths (1,306) was noted from 2019 to 2024, whereas the lowest number (914) occurred from 2004 to 2008.

**Table 2:** Overall mortality due to vaccine-preventable diseases among children (<18 years). Crude mortality rate is expressed per 100,000 population. Population data were obtained from CDC WONDER. CDC WONDER: Centers for Disease Control and Prevention Wide-ranging Online Data for Epidemiologic Research

Study period	Deaths (n)	Population	Crude mortality rate (per 100,000)
1999-2003	1,202	362,948,251	0.33
2004-2008	914	368,703,125	0.25
2009-2013	1,137	369,563,866	0.31
2014-2018	961	367,925,734	0.26
2019-2024	1,306	437,843,104	0.30
Overall	5,520	1,906,984,080	0.29

Table 3 shows the demographic characteristics of 5,520 deaths due to preventable diseases in children under 18 years from 1999 to 2024. Infants under one year of age had the highest percentage of deaths (n=1,582; 28.7%), followed by one- to four-year-old children (n=1,553; 28.1%), while the lowest percentage was among those aged 15-17 years (n=526; 9.5%). Overall, males accounted for a slightly higher proportion of deaths compared to females (52.5% vs. 47.5%). By race, the majority of deaths happened among White children (71.0%), while the second largest number of deaths was recorded among Black or African American children (20.3%). By region, the highest percentage of deaths was seen in the South region (38.8%), followed by the West (26.0%), the Midwest (21.7%), and the Northeast (13.5%). The majority of deaths took place in large central metropolitan areas (30.5%), while non-core (non-metro) areas accounted for the smallest proportion (7.8%).

**Table 3:** Baseline characteristics of vaccine-preventable disease mortality among children. AI: American Indian; AN: Alaska Native

Characteristics	Category	Deaths (n)	%
Age group	<1 year	1,582	28.7
	1-4 years	1,553	28.1
	5-9 years	1,001	18.1
	10-14 years	858	15.5
	15-17 years	526	9.5
	Total	5,520	100
Sex	Female	2,622	47.5
	Male	2,898	52.5
	Total	5,520	100
Race	White	3,919	71
	Black or African American	1,120	20.3
	Asian or Pacific Islander	260	4.7
	AI or AN	221	4
	Total	5,520	100
Region	Northeast	747	13.5
	Midwest	1,199	21.7
	South	2,139	38.8
	West	1,435	26
	Total	5,520	100
Urban-rural classification	Large central metro	1,685	30.5
	Medium metro	1,242	22.5
	Large fringe metro	1,090	19.7
	Micropolitan (non-metro)	551	10
	Small metro	520	9.4
	Non-core (non-metro)	432	7.8
	Total	5,520	100

Mortality from vaccine-preventable diseases for disease-specific causes in children aged <18 years is provided in Table 4 from 1999 to 2024. The highest cause of death was influenza (n=3,352; 60.7%), followed by meningococcal disease (n=783; 14.2%), and pneumococcal disease (n=646; 11.7%). Pertussis caused 6.1%, hepatitis A 3.2%, and *Haemophilus influenzae* disease 2.9% of the deaths, while only 1.2% of deaths were attributed to varicella. The data for measles and tetanus were suppressed due to small numbers, while there were no deaths from mumps, rubella, or diphtheria. The overall crude mortality rate was 0.29 per 100,000 children (Table 4).

**Table 4:** Disease-specific mortality from vaccine-preventable diseases among children.

Disease	Deaths (n)	%	Crude mortality rate (per 100,000)
Influenza	3,352	60.7	0.15
Meningococcal disease	783	14.2	0.04
Pneumococcal disease	646	11.7	0.03
Pertussis (whooping cough)	338	6.1	0.02
Hepatitis A	176	3.2	0.01
*Haemophilus influenzae* disease	160	2.9	0.01
Varicella	65	1.2	<0.01
Measles	Suppressed	-	Suppressed
Tetanus	Suppressed	-	Suppressed
Mumps	0	0.0	0.0
Rubella	0	0.0	0.0
Diphtheria	0	0.0	0.0
Total	5,520	100	0.29

Table 5 represents the Joinpoint regression analysis of the temporal trends for mortality associated with disease-specific vaccine-preventable diseases in children <18 years old. Mortality due to influenza revealed a statistically significant increasing trend over the time period 1999-2024 (APC=5.02%, 95% CI: 0.78 to 9.27; p=0.020). On the other hand, pneumococcal disease mortality decreased significantly from 1999 to 2011 (APC = -10.64%; p<0.001) and remained stable until 2024. Mortality due to pertussis revealed a non-significant decreasing trend over the study period (APC = -1.27%; p=0.248). Mortality due to meningococcal diseases revealed a statistically significant decreasing trend over 2010-2013 (APC = -23.37%; p=0.020), while the remaining segments showed no statistically significant changes (Table 5).

**Table 5:** Joinpoint regression analysis of temporal trends in disease-specific mortality from vaccine-preventable diseases among children. Disease-specific mortality trends were analyzed using Joinpoint regression with the Monte Carlo permutation test. APCs, 95% CIs, and p-values are reported. Each segment represents a period with a distinct linear trend identified by the Joinpoint regression model. APC: annual percent change

Disease	Segment	Study period	APC (%)	95% CI	p-Value
Influenza	1	1999-2024	5.02	0.78 to 9.27	0.020
Pneumococcal disease	1	1999-2011	-10.64	-27.63 to -6.61	<0.001
	2	2011-2024	0.14	-4.01 to 32.17	0.791
Pertussis (whooping cough)	1	1999-2024	-1.27	-3.54 to 0.90	0.248
Meningococcal disease	1	1999-2010	-11.54	-15.16 to 12.26	0.075
	2	2010-2013	-23.37	-30.42 to -11.10	0.020
	3	2013-2024	-0.96	-6.27 to 5.73	0.817

Table 6 summarizes the Joinpoint regression analysis for age-specific mortality trends for children <18 years old. There was a significantly decreasing trend in infant deaths (<1 year) during 1999 through 2024 (APC = -3.10%, 95% CI: -4.71 to -1.44; p<0.001), while there was a significantly increasing trend among five- to nine-year-old children (APC=3.42%, 95% CI: 0.54-6.37; p=0.020). There were no significantly changing trends for children one- to four-year-old (APC=0.38%; p=0.759) or 10-14 years (APC=1.58%; p=0.215).

**Table 6:** Joinpoint regression analysis of age-specific mortality trends among children. APCs, 95% CIs, and corresponding p-values were estimated using Joinpoint regression analysis with the Monte Carlo permutation test for age-specific mortality trends among children (<18 years) in the United States from 1999 to 2024. Each segment represents a distinct linear trend identified by the Joinpoint regression model. A positive APC indicates an increasing mortality trend, whereas a negative APC indicates a decreasing mortality trend. A p<0.05 was considered statistically significant. APC: annual percent change

Age group	Segment	Study period	APC (%)	95% CI	p-Value
<1 year	1	1999-2024	-3.10	-4.71 to -1.44	<0.001
1-4 years	1	1999-2024	0.38	-2.36 to 3.16	0.759
5-9 years	1	1999-2024	3.42	0.54 to 6.37	0.020
10-14 years	1	1999-2024	1.58	-0.93 to 4.12	0.215

The distribution of disease-specific mortality over the course of the study period is illustrated in Table 7. Influenza mortality increased over time, rising from 330 deaths during 1999-2003 to 1,065 during 2019-2024. On the other hand, mortality due to meningococcal and pneumococcal diseases decreased significantly over successive time periods, with meningococcal deaths reducing from 328 to 20 and pneumococcal deaths from 257 to 64. Pertussis mortality remained relatively low for the entire study period. However, deaths due to varicella, hepatitis A, and *Haemophilus influenzae* type b (Hib) infections were low and occurred mostly during the early periods of the study period. The association between study period and disease-specific mortality was statistically significant (p<0.001).

**Table 7:** Association between study period and disease-specific mortality due to vaccine-preventable infectious diseases. Symbol (-) indicates a suppressed value in the CDC WONDER database due to a small number of deaths. Overall values represent the total deaths during the study period. Pearson's chi-square test was used to compare the distribution of disease-specific mortality across study periods (χ^2^=1563.56, df=24, p<0.001). Hib: *Haemophilus influenzae* type b; χ^2^: Pearson's chi-square statistic; CDC WONDER: Centers for Disease Control and Prevention Wide-ranging Online Data for Epidemiologic Research

Study period	Influenza	Meningococcal	Pneumococcal	Pertussis	Varicella	Hepatitis A	Hib	*χ*^2^ value	p-Value
1999-2003	330	328	257	77	30	87	23	1563.56	<0.001
2004-2008	409	203	140	67	-	11	13		
2009-2013	809	105	90	73	-	-	-		
2014-2018	739	44	67	12	-	-	-		
2019-2024	1,065	20	64	25	-	11	21		
Overall	3,352	783	646	338	65	176	160		

**Figure 2:**
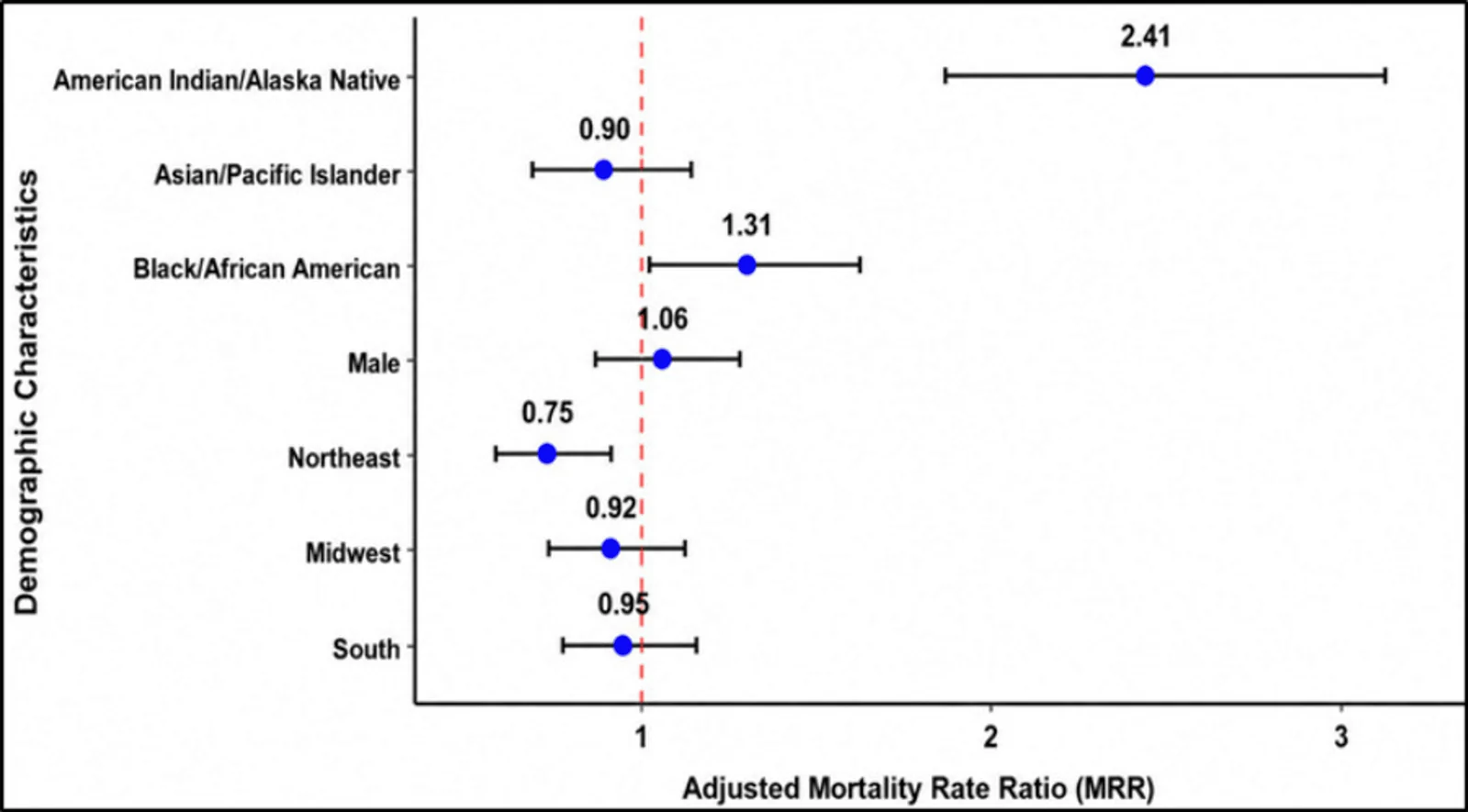
Forest plot of adjusted mortality rate ratios from negative binomial regression. Forest plot showing adjusted mortality rate ratios (MRRs) and 95% confidence intervals (CIs) for demographic factors associated with vaccine-preventable disease mortality among children (<18 years) in the United States, 1999-2024. The blue circles represent the adjusted MRR estimates, and the horizontal lines indicate the corresponding 95% CIs. The red dashed vertical line at MRR represents the reference value, indicating no difference in mortality risk relative to the reference category. Estimates with 95% CIs that do not cross one are considered statistically significant.

Negative binomial regression analysis identified significant racial disparities in vaccine-preventable disease mortality. Compared with White children (reference category), AI/AN children had a 2.41-fold higher adjusted mortality rate (mortality rate ratio {MRR}=2.41, 95% CI: 1.87-3.10; p<0.001), while Black/African American children had a 1.31-fold higher adjusted mortality rate (MRR=1.31, 95% CI: 1.05-1.63; p=0.017). No significant associations were observed for Asian/Pacific Islander children or sex in the adjusted negative binomial regression analysis (Table 8). Regionally, children in the Northeast had a significantly lower adjusted mortality rate than those in the West (MRR=0.75, 95% CI: 0.61-0.92; p=0.006), whereas mortality rates in the Midwest and South did not differ significantly from those in the West (Table 8 and Figure 2).

**Table 8:** Negative binomial regression analysis of demographic factors associated with vaccine-preventable disease mortality. An MRR >1 indicates a higher mortality rate relative to the reference category, whereas an MRR <1 indicates a lower mortality rate. A p<0.05 was considered statistically significant. Negative binomial regression results are presented as MRRs with 95% CIs. Wald z-statistics and corresponding p-values are reported for each predictor. MRR: mortality rate ratio; AI: American Indian; AN: Alaska Native

Variables	Category (reference)	MRR	95% CI	Wald *z*	p-Value
Race	AI/AN (White)	2.41	1.87-3.10	6.87	<0.001
	Asian/Pacific Islander (White)	0.90	0.70-1.15	-0.826	0.409
	Black/African American (White)	1.31	1.05-1.63	2.40	0.017
Sex	Male (female)	1.06	0.87-1.28	0.552	0.581
Region	Northeast (West)	0.75	0.61-0.92	-2.740	0.006
	Midwest (West)	0.92	0.75-1.13	-0.795	0.426
	South (West)	0.95	0.78-1.16	-0.502	0.616

## Discussion

This nationwide retrospective population-based study analyzed 5,520 deaths due to VPIDs among children under 18 years of age in the United States (1999-2024). The overall crude mortality rate has only marginally reduced from 0.33 per 100,000 (1999-2003) to 0.25 per 100,000 (2004-2008) and has oscillated around that level since 2008, reaching 0.30 per 100,000 (2019-2024). Influenza was the predominant cause of VPID deaths (60.7%) and was significantly increasing at a rate of 5.02% per year (95% CI: 0.78-9.27; p=0.020). Mortality from meningococcal and pneumococcal disease declined significantly following the introduction of the conjugate vaccines; mortality for varicella, *Haemophilus influenzae* type b (Hib), and hepatitis A also reduced significantly. The number of children who died in infancy significantly declined (APC = -3.10%, p<0.001), while deaths among children aged five to nine years significantly increased (APC=3.42%, p=0.020). AI/AN children had the largest racial disparity (mortality rate ratio {MRR}=2.41; 95% CI: 1.87-3.10), followed by Black/African American children (MRR=1.31; 95% CI: 1.05-1.63). This stood in stark contrast to the Northeast, which had the lowest mortality (13.5%) and the South, with the highest mortality (38.8%).

The fluctuating pattern in VPID mortality seen in the last 25 years aligns with the pattern seen worldwide. The Global Burden of Disease (GBD) Study 2023 revealed significant reductions in communicable disease burden between 2010 and 2023, but warned that this progress may be lost or even reversed unless continued funding for vaccination and public health systems is provided [14]. Notably, in 2023, routine childhood vaccine coverage for several vaccine-dose combinations was not up to pre-pandemic levels globally, as estimated by GBD 2023. Causal relationships cannot be drawn from the present ecological analysis, yet these persistent coverage gaps may have played a role in the observed temporal mortality patterns from 2019 to 2024 [2].

The increasing influenza mortality observed in our study is consistent with FluSurv-NET surveillance, which shows that severe influenza continues to be an important cause of pediatric disease. Seasonal variation in pediatric influenza-associated hospitalization rates was also significant between 2010 and 2023, with higher age-adjusted rates of influenza hospitalization among Black/African American and AI/AN populations in 2010-2023, similar to that seen in our mortality analysis [15].

Several factors have contributed to the continued rise in influenza mortality as follows: low vaccine coverage, mismatch between influenza strains and the vaccine, and heightened influenza activity following two seasons of suppressed circulation during the COVID-19 pandemic due to reduced population immunity. While Russo et al. focused their study on older adults (aged ≥65 years), they also found that influenza-related gains during the pandemic fell away in the following seasons, with likely parallels in the pediatric setting [16]. The significant increase in VPID mortality among children aged five to nine years (APC=3.42%, p=0.020) may, in part, reflect the increasing burden of influenza in this age group. School-aged children are recognized as important contributors to influenza transmission because of frequent close contact in school settings, while inconsistent seasonal influenza vaccination coverage may have also contributed to the observed trend.

The pneumococcal decline is consistent with the systematic review by Reyburn et al. that showed that pneumococcal conjugate vaccines lowered pneumonia hospitalization rates by 7-60% and pneumonia mortality rates by 10-78% in children aged 0 to nine years [17]. In the rest of the world, Wahl et al. estimated a 51% and 90% decrease in pneumococcal and Hib deaths, respectively, among children aged one to 59 months between 2000 and 2015, which mirrors the domestic pneumococcal and Hib-specific trends observed here [18].

The marked decrease in pneumococcal mortality between 1999 and 2011 (APC = -10.64%, p<0.001) supports the temporal association with the introduction of the seven-valent pneumococcal conjugate vaccine (PCV7) in 2000, followed by the 13-valent pneumococcal conjugate vaccine (PCV13) in 2010. The lack of statistical significance of the trend thereafter (APC=0.14%, p=0.791) could be due to the continuing disease burden from non-vaccine serotypes and changing pneumococcal epidemiology, highlighting the need for continued monitoring. Similarly, the APC for meningococcal mortality from 2010 to 2013 was the largest negative change (APC = -23.37%; p=0.020), which is likely due to the implementation of routine MenACWY vaccination in 2005 and the introduction of the booster dose at 16 years of age in 2010. Although meningococcal mortality has remained low since then, it suggests that the disease is not eradicated as a vaccine-preventable disease. Meningococcal vaccines for MenB were introduced in 2014-2015 with a shared clinical decision-making approach, and uptake of these vaccines among older adolescents continues to be suboptimal, due in part to the challenges of shared clinical decision-making, as well as provider recommendations for vaccination [19].

There was a non-significant decreasing trend in pertussis mortality (APC = -1.27%, p=0.248), and deaths remained high among infants who were too young to have received the full DTaP vaccination course. Maternal Tdap vaccination is considered the most effective strategy to provide these vulnerable infants with protection before their eligibility for routine immunization via transplacental antibody transfer. The study by Baïssas et al. also emphasized the need for effective immunization programs and continued engagement with healthcare providers to achieve high maternal vaccination rates. While progress in infant vaccine-preventable disease mortality was significant in our study (APC = -3.10%, p<0.001), this may be because of several improvements in childhood disease prevention, such as improved immunization programs for mothers and infants [20].

Influenza, the only significantly rising vaccine-preventable disease in this study, indicates the necessity of reinforcing annual vaccination programs against influenza in childhood. Doxey et al. found the risk of influenza death twice as high in AI/AN aged under five years compared to non-Hispanic Whites in the period of 1999-2016 and stressed the issue of racial disparity in terms of influenza mortality and the necessity of targeted public health interventions because these deaths are largely vaccine-preventable [21]. According to Jarshaw et al., Black respondents had the lowest rate of prenatal influenza vaccination uptake among other ethnicities (44.4%), which was likely to lead to increased infant mortality due to lack of maternal antibodies [22]. Therefore, it should be noted that mere availability of vaccines does not suffice, as one must tackle such barriers as poverty, healthcare inequality, and institutional distrust. Finally, the geographical distribution (MRR of 0.75 in the Northeast region vs. the Western region; 38.8% of deaths occurred in the South) suggests implementing different immunization strategies depending on the region. Catch-up vaccination programs can be considered necessary, considering that global childhood vaccination coverage did not reach pre-pandemic levels by 2023 [2].

In contrast, no significant associations were observed for Asian/Pacific Islander children or sex after adjustment. This may reflect comparatively lower mortality rates, smaller numbers of deaths, or limited statistical power to detect differences within these subgroups. However, these findings should be interpreted cautiously, and further studies incorporating individual-level clinical and socioeconomic data are needed to better understand these associations.

Strengths and limitations

The strengths of this study are its nationally representative 25-year dataset, the use of Joinpoint regression for characterizing trends, and the use of negative binomial regression for handling overdispersion. Several limitations need to be considered. First, there may have been misclassification or underreporting of the cause of death because of the use of death certificate data, especially for influenza. Second, due to cell suppression in the CDC WONDER database when fewer than 10 deaths occur in certain age groups, formal analysis was not possible for some low-frequency diseases like measles and tetanus. Third, the lack of information regarding individual-level vaccination status, socioeconomic factors, and access to care did not allow for adjustment of potential confounders, and causality could not be ascertained. Fourth, the racial misclassification on death certificates may have resulted in underestimation of mortality among children [21]. Additionally, although overlapping years between the CDC WONDER bridged-race (1999-2020) and single-race (2018-2024) databases were handled using non-overlapping extraction periods to avoid duplicate records, differences in race classification methods and coding practices between the two databases may have introduced minor inconsistencies in mortality estimates over time. Finally, due to the ecological design, this study cannot establish causal relationships between vaccination programs/policies and mortality trends.

Future recommendations

For future research, individual-level vaccination status and socioeconomic characteristics, in addition to mortality rates, should be combined to differentiate between vaccine coverage rates and structural obstacles to access to vaccination. Research on the consequences of COVID-19-related disruptions to vaccination efforts on VPID mortality in 2024-2030 is recommended, as global routine childhood vaccination coverage had not returned to pre-COVID levels by 2023 [2]. Prospective surveillance of meningococcal and pneumococcal diseases, including their serotypes, should be conducted to monitor serotype replacement. Targeted intervention research addressing structural determinants of influenza vaccination disparities in AI/AN and Black/African American children remains a priority.

## Conclusions

The findings from this nationwide retrospective study demonstrate that, despite substantial progress in childhood immunization, mortality due to vaccine-preventable infectious diseases remains an important public health concern. Although mortality declined for most vaccine-preventable diseases, influenza remained the leading cause of death and the only vaccine-preventable disease to demonstrate an increasing mortality trend during the study period. Persistent demographic and geographic disparities further suggest that the benefits of immunization have not been achieved equitably across all populations.

These findings highlight the importance of strengthening routine and catch-up immunization programs, improving equitable access to vaccination services, and maintaining continuous mortality surveillance. Although causal relationships cannot be inferred from this ecological study, these measures may help address persistent disparities in vaccine-preventable infectious disease mortality and guide future public health strategies. Continued investment in evidence-based immunization strategies and targeted public health interventions will be essential to further reduce preventable childhood deaths and sustain the long-term gains achieved through vaccination.
